# Positive staining of the immunoligand B7-H6 in abnormal/transformed keratinocytes consistently accompanies the progression of cervical cancer

**DOI:** 10.1186/s12865-020-0341-9

**Published:** 2020-03-06

**Authors:** Gloria Yareli Gutierrez-Silerio, Ramon Antonio Franco-Topete, Jesse Haramati, Eduardo Miguel Navarrete-Medina, Jorge Gutierrez-Franco, Miriam Ruth Bueno-Topete, Blanca Estela Bastidas-Ramirez, Martha Eloisa Ramos-Marquez, Susana del Toro-Arreola

**Affiliations:** 1 Instituto de Enfermedades Crónico Degenerativas, Departamento de Biología Molecular y Genómica, CUCS, Universidad de Guadalajara, Sierra Mojada # 950, Colonia Independencia, CP 44340 Guadalajara, Jalisco Mexico; 2 Instituto Transdisciplinar de Investigación y Servicios, CUCEI, Universidad de Guadalajara, Guadalajara, Jalisco Mexico; 3 Laboratorio de Patología, Departamento de Microbiología y Patología, CUCS, Universidad de Guadalajara, Guadalajara, Jalisco Mexico; 4 Departamento de Anatomía Patológica, Nuevo Hospital Civil de Guadalajara “Dr. Juan I. Menchaca”, Guadalajara, Jalisco Mexico; 5 Laboratorio de Inmunobiología, Departamento de Biología Celular y Molecular, CUCBA, Universidad de Guadalajara, Guadalajara, Jalisco Mexico; 6 Unidad Académica de Ciencias Químico Biológicas y Farmacéuticas, Universidad Autónoma de Nayarit, Tepic, Nayarit Mexico; 7 Laboratorio de Inmunología, Departamento de Fisiología, CUCS, Universidad de Guadalajara, Guadalajara, Jalisco Mexico

**Keywords:** B7-H6, B7H6, Cervical cancer, Cervical intraepithelial lesions, Therapeutic target, NKp30

## Abstract

**Background:**

B7-H6 has been revealed as an endogenous immunoligand expressed in a variety of tumors, but not expressed in healthy tissues. Heretofore, no studies have been reported describing B7-H6 in women with cervical cancer. To investigate this question, our present study was conducted.

**Results:**

This retrospective study comprised a total of 62 paraffinized cervical biopsies, which were distributed in five groups: low-grade squamous intraepithelial lesions (LSIL), high-grade squamous intraepithelial lesions (HSIL), squamous cervical carcinoma (SCC), uterine cervical adenocarcinoma (UCAC), and a group of cervicitis (as a control for non-abnormal/non-transformed cells). Cervical sections were stained by immunohistochemistry to explore the expression of B7-H6, which was reported according to the immunoreactive score (IRS) system. We observed a complete lack of B7-H6 in LSIL abnormal epithelial cells. Interestingly, B7-H6 began to be seen in HSIL abnormal epithelial cells; more than half of this group had B7-H6 positive cells, with staining characterized by a cytoplasmic and membranous pattern. B7-H6 in the SCC group was also seen in the majority of the sections, showing the same cytoplasmic and membranous pattern. Strong evidence of B7-H6 was notably found in UCAC tumor columnar cells (in 100% of the specimens, also with cytoplasmic and membranous pattern). Moreover, consistent B7-H6 staining was observed in infiltrating plasma cells in all groups.

**Conclusions:**

B7-H6 IRS positively correlated with disease stage in the development of cervical cancer; additionally, B7-H6 scores were found to be even higher in the more aggressive uterine cervical adenocarcinoma, suggesting a possible future therapeutic target for this cancer type.

## Background

Cervical cancer is the fourth most common cancer in women worldwide. Specifically in Mexico, as of 2018, this cancer is the third most common tumor in terms of incidence and mortality in the female population [1].

The development of cervical cancer has been reported as a sequence of steps, defined through histopathological classifications, starting with high-risk human papillomavirus (HPV) infection, low-grade squamous intraepithelial lesion (LSIL), high-grade squamous intraepithelial lesion (HSIL), and finally invasive cervical cancer. Although the incidence of HPV infection is widespread within young sexually active women, more than 90% of those infected will typically clear HPV within one to 3 years of detection. However, the long-term persistence of high-risk HPV infection can eventually lead to the progression of cervical cancer [2, 3].

The two most prominent cervical cancer types, classified according to their histology, are squamous cervical carcinoma (SCC) and uterine cervical adenocarcinoma (UCAC). Approximately 75–90% of the cervical tumors are squamous cervical carcinomas, and the remaining 10–25% are uterine cervical adenocarcinomas. Despite the overwhelming prevalence of HPV infections in women, the actual incidence of tumor development in women infected with or without lesions remains very low. There are, clearly, other factors implicated in the progression of the disease, such as immune status and function [4–6]. It follows, then, that novel or emerging treatments of cervical cancer could be addressed through the identification of unique markers, such as stress ligands expressed by tumors, which could lead to immune system activation, or rather immune failure via immune escape mechanisms.

One major role of the immune system is the surveillance against tumors; for instance, NK cells inspect autologous cell surfaces for aberrant expression of MHC class I molecules and stress molecules that have emerged as ligands of activating receptors, such as NKG2D, NKp46, NKp44, or NKp30, which will promote the NK cell cytotoxic activity [7]. In particular, NKp30 has been found to be significantly diminished in patients with high-grade squamous intraepithelial lesions, as well as with cervical cancer [8].

The identification of NKp30 ligands has been a great challenge, and to date little is known about this topic. However, in the last decade, the molecule B7-H6 has been revealed as a novel NKp30 endogenous immunoligand expressed in a variety of tumors and stressed cells, but not expressed in healthy tissues [9–14].

Due to the fact that B7-H6 belongs to a family composed of both co-stimulatory and co-inhibitory receptors [14], it follows that the supposition has arisen that this ligand for NKp30 also may play a role in the biology of the tumor cells themselves, perhaps promoting tumor cell survival, proliferation, migration and invasion [9, 15, 16]. Cell culture studies have shown that the interaction between NKp30/B7-H6 drives a signaling cascade in the NK cell that culminates in degranulation and IFN-γ secretion [14]. However, some in vivo studies show an opposite effect: in these experiments, high cell surface or soluble expression of B7-H6 were associated with dysfunction of NK cells, which was manifested as decreased cytotoxicity and down-regulation of NKp30 [11, 17, 18].

At present, the precise expression pattern of B7-H6 has only been partially elucidated in some tumors; whether B7-H6 is expressed during the development of cervical cancer still remains unknown. Thus, the goal of this study was to explore the presence of B7-H6 in the cervix of women with cervicitis (as a control for non-abnormal/non-transformed cells), LSIL, HSIL, SCC, and UCAC.

## Results

### B7-H6 expression in abnormal/transformed cells

**Fig. 1:**
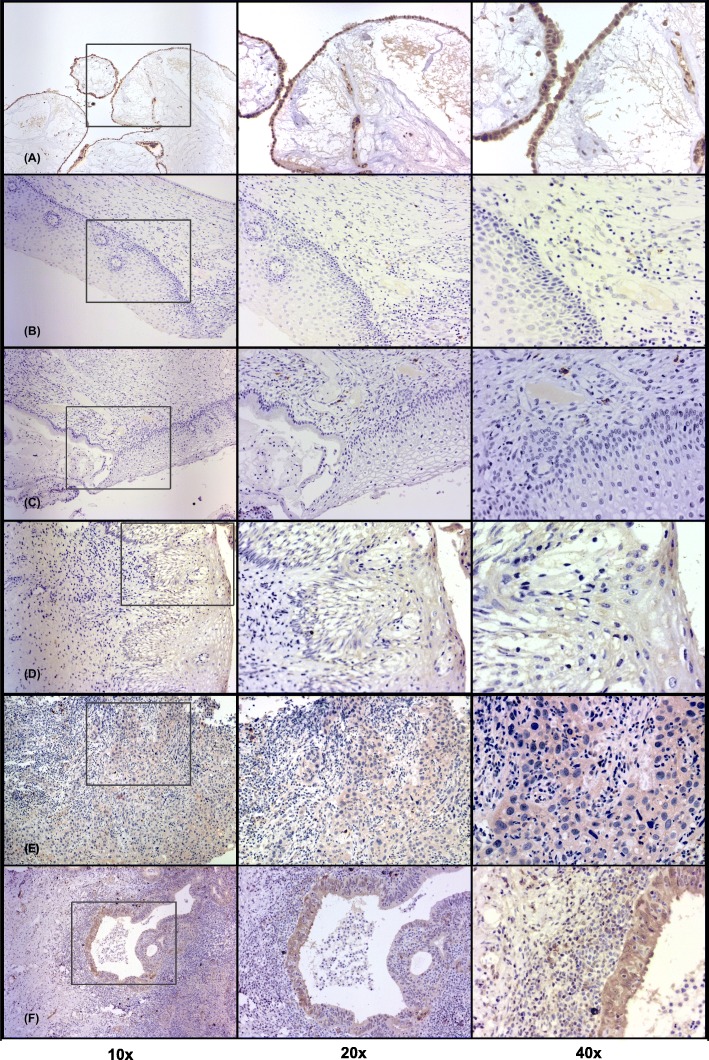
Representative immunohistochemical staining pattern of B7-H6 in cervical precancerous lesions and invasive cervical cancer. Specimens were categorized as follows: (**a**) ovarian cystadenoma (as the positive control): B7-H6 is mainly expressed in lining epithelium; (**b**) cervicitis: absence of B7-H6 in keratinocytes; (**c**) LSIL: absence of B7-H6 in abnormal keratinocytes; (**d**) HSIL: presence of B7-H6 in abnormal keratinocytes; (**e**) SCC: B7-H6 expression in transformed tumoral cells; (**f**) UCAC: strong expression of B7-H6 in transformed tumoral cells. Positive staining of B7-H6 is seen in the cytoplasm and membrane of transformed cells, and in plasma cells and mononuclear cells infiltrating the stroma. Photomicrographs were taken with different objectives (10x, 20x, and 40x). *Abbreviations: LSIL: low-grade squamous intraepithelial lesion; HSIL: high-grade squamous intraepithelial lesion; SCC: squamous cervical carcinoma; UCAC: uterine cervical adenocarcinoma*

With the aim of elucidating whether B7-H6 is expressed in dysplastic cervical lesions or in cervical cancer, we performed B7-H6 immunohistochemistry staining analyses. The tissue of an ovarian cystadenoma was used as a positive control (Fig. 1a), which is B7-H6 positive, according to previous reports [19].

First, in order to have the closest to what we considered a representative control, we included biopsies of the cervix removed for causes other than cervical intraepithelial lesions or cervical cancer (all of these “control” samples presented cervicitis). It is important to note that there were no abnormal or transformed cells in the specimens of the cervicitis group (no HPV infection reported) (Fig. 1b). The patients in this group presented an inflammatory condition that clearly could not be considered as a healthy cervix. These specimens were obtained from biopsies of the cervix removed due to different causes, such as leiomyomas or, in a few cases, from prolapsed bladder surgery. We observed B7-H6 positivity in 3 out of the 10 specimens of the cervicitis group; importantly, B7-H6 expression was located in a distinctive manner (parabasal layer staining) and presented a median IRS of 0. Of note is the fact that the highest score was found in an atypical specimen with acute inflammation (data not shown), implying that B7-H6 staining here may be correlated with the level of local inflammation.

**Fig. 2:**
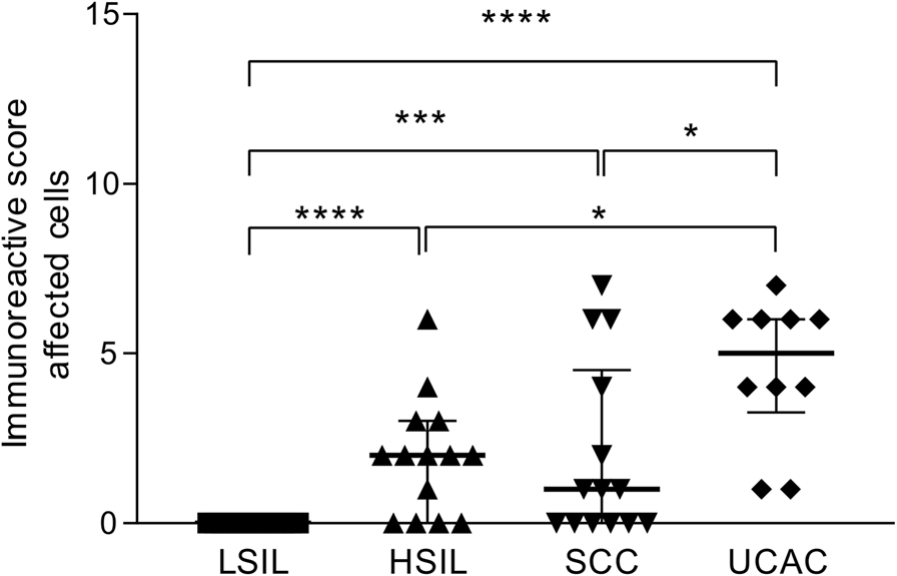
B7-H6 positivity according to the immunoreactive score in abnormal/transformed cells in cervical precancerous lesions and invasive cervical cancer. The results show a strong positive correlation coefficient between B7-H6 positivity and stage of the disease (LSIL, HSIL, and SCC) (ρ = 0.456), and a significance of *p* = 0.002. HSIL and both cervical cancer types present significant statistical differences when compared against LSIL. HSIL vs LSIL (*p* < 0.0005), SCC vs LSIL (*p* = 0.001) and UCAC vs LSIL (p < 0.0005). No significant differences were found between HSIL and SCC scores, but significance was found when comparing HSIL vs UCAC (*p* = 0.008), and SCC vs UCAC (*p* = 0.023). *Abbreviations: LSIL: low-grade squamous intraepithelial lesion; HSIL: high-grade squamous intraepithelial lesion; SCC: squamous cervical carcinoma; UCAC: uterine cervical adenocarcinoma.* ****p < 0.0005, ***p = 0.001, *p < 0.05

We observed a complete lack of expression of B7-H6 in abnormal epithelial cells in all of the low-grade squamous intraepithelial lesion (LSIL) specimens analyzed (a representative stained specimen is depicted in Fig. 1c and summarized in Fig. 2). The positivity for B7-H6 begins to be seen in abnormal epithelial cells of the high-grade squamous intraepithelial lesion (HSIL) group; this was the case in 10 out of the 14 HSIL samples (71%). The staining pattern seen was characterized as cytoplasmic and membranous (Fig. 1d). The median immunoreactive score (IRS) for this group was 2 (Fig. 2).

Similar to the HSIL group, in the squamous cervical carcinoma (SCC) group we found that B7-H6 is expressed in the majority of specimens, 9/14 (64%), with the same cytoplasmic and membranous pattern (Fig. 1e), with a median IRS of 1 (Fig. 2). Statistically, there was no difference between HSIL and SCC groups. Nevertheless, when compared against the LSIL group, there was a statistically significant difference vs HSIL (*p* < 0.0005) and vs SCC (*p* = 0.001), as summarized in Fig. 2.

Interestingly, strong evidence of the presence of B7-H6 was found in tumor columnar cells of the uterine cervical adenocarcinoma (UCAC) specimens, inasmuch as 100% of the specimens were positive with the same cytoplasmic and membranous pattern (representative stained sample is shown in Fig. 1f). These samples also presented a higher intensity than the aforementioned groups, with a median IRS of 4.5 (Fig. 2). Indeed, when compared against the different groups, there is a significant difference between UCAC vs LSIL (*p* < 0.0005), UCAC vs HSIL (*p* = 0.008), and UCAC vs SCC (*p* = 0.023).

Due to the above-mentioned results, a Spearman correlation test was applied between the different progressive groups (LSIL, HSIL, and SCC) and the B7-H6 positivity seen in abnormal/transformed cells. The results show a strong positive correlation coefficient (ρ = 0.456), with a significance of *p* = 0.002, suggesting that B7-H6 expression correlates with the stage of the disease.

### B7-H6 in inflammatory infiltrating cells: plasma cells and other mononuclear cells

**Fig. 3:**
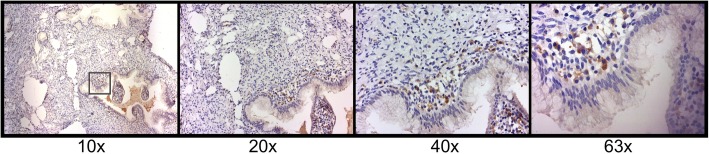
B7-H6 immunoreactivity in plasma cells. B7-H6 positivity shows a cytoplasmic and membranous pattern in plasma cells. Photomicrographs were taken with different objectives (10x, 20x, 40x, and 63x) from a cervicitis sample. Positivity in plasma cells was considered as an internal control for the evaluation of B7-H6 staining in other cells

Another important finding to result from this study is that B7-H6 expression was also found in some mononuclear cells infiltrating the stroma; this finding was more notable in those cells morphologically identified as plasma cells. The positive staining observed in these cells was notably consistent and used as an internal control for our experimental conditions (Fig. 3).

**Fig. 4:**
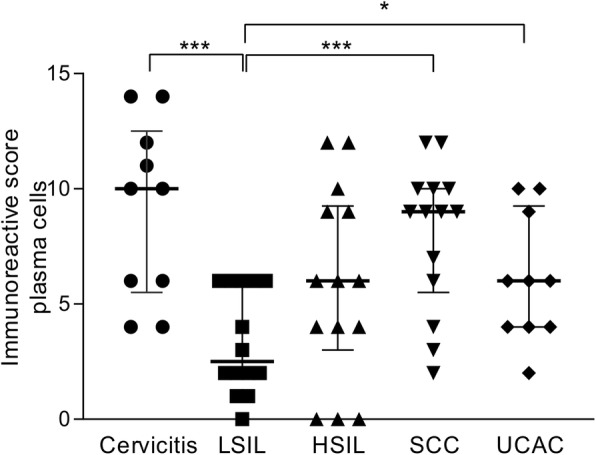
B7-H6 positive staining according to the immunoreactive score in plasma cells infiltrating cervical intraepithelial lesions or invasive cancer. B7-H6 positivity in plasma cells is positively correlated with disease stage, confirmed by low staining in the intraepithelial lesion groups and stronger B7-H6 staining in the cancer groups. Statistically significant differences when comparing LSIL vs SCC (p = 0.001) and LSIL vs UCAC (*p* = 0.026) were found. As a control of non-abnormal or non-transformed cells, cervicitis specimens were also assessed. This group showed higher B7-H6 positive staining when compared against LSIL (p = 0.001). A positive correlation of B7-H6 staining between plasma cells and stage of the disease (LSIL, HSIL, and SCC) was found with ρ = 0.515 and a significance of *p* < 0.0005. *Abbreviations: LSIL: low-grade squamous intraepithelial lesion; HSIL: high-grade squamous intraepithelial lesion; SCC: squamous cervical carcinoma; UCAC: uterine cervical adenocarcinoma.* ***p = 0.001, *p < 0.05

Interestingly, B7-H6 positive staining (showing both cytoplasmic and membranous staining patterns) was observed in infiltrating plasma cells in all groups. Looking deeper into the different groups, we saw a higher expression of B7-H6 in the cervicitis group when compared against the LSIL group (*p* = 0.001) (Fig. 4). Between the cervical intraepithelial lesions or cervical cancers we found an interesting pattern, where the positive staining of B7-H6 was found in a higher percentage and intensity in infiltrating plasma cells in both types of cervical cancer. Our results showed a statistically significant difference when comparing LSIL vs SCC (p = 0.001) and LSIL vs UCAC (*p* = 0.026) (Fig. 4). Moreover, we also found a strong positive Spearman correlation (ρ = 0.515, *p* < 0.0005) between plasma cells IRS and stage of the disease (LSIL, HSIL, and SCC).

**Fig. 5:**
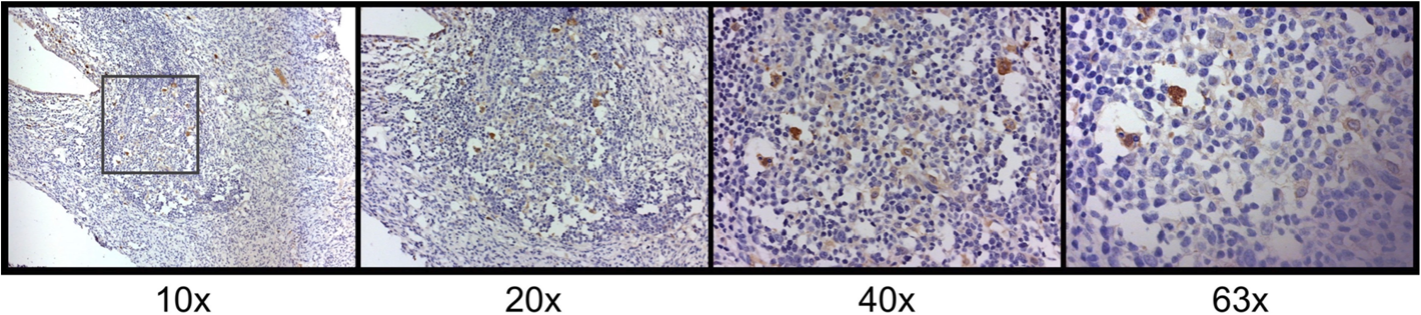
B7-H6 staining in mononuclear cells. All mononuclear cells with positive staining show cytoplasmic and membranous patterns. There are no differences in IRS, among the groups analyzed. Photomicrographs were taken with different objectives (10x, 20x, 40x, and 63x) from a chronic follicular cervicitis sample

**Fig. 6:**
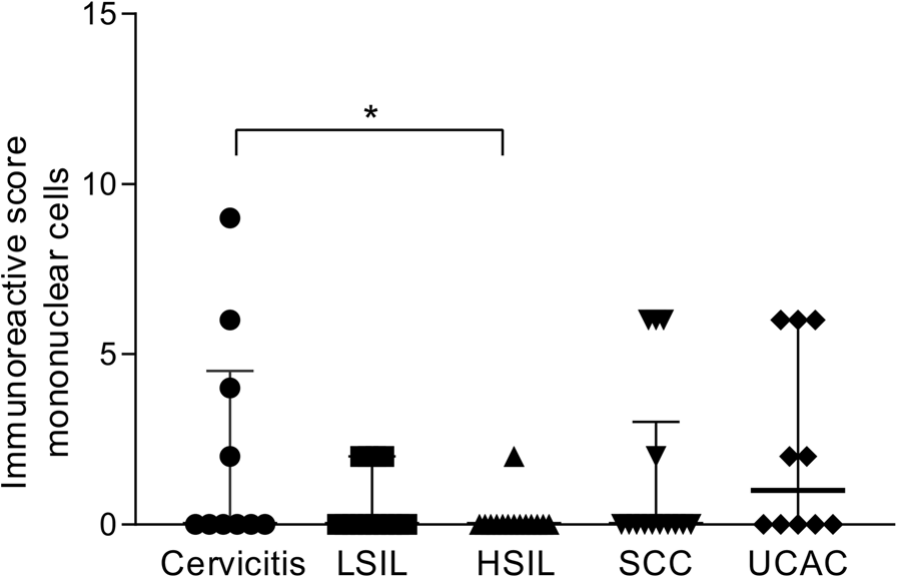
B7-H6 positive staining according to the immunoreactive score in mononuclear cells infiltrating cervical intraepithelial lesions or invasive cancer. Immunoreactivity for B7-H6 is higher in mononuclear cells of the cervicitis group, compared with the HSIL group (*p* = 0.043). Cervicitis specimens were used as control for non-abnormal or non-transformed cells. *Abbreviations: LSIL: low-grade squamous intraepithelial lesion; HSIL: high-grade squamous intraepithelial lesion; SCC: squamous cervical carcinoma; UCAC: uterine cervical adenocarcinoma.* *p < 0.05

Additionally, we also observed B7-H6 positive staining in other mononuclear cells infiltrating the stroma. We propose macrophages, dendritic cells and T lymphocytes as possible B7-H6 positive cells; though, this finding will require a deeper analysis in order to determine with certainty the identity of these cells. Nevertheless, despite not knowing the cell lineage of these populations, no differences regarding B7-H6 positive infiltrating mononuclear cells were observed between cervical intraepithelial lesions or cervical cancer groups. However, when comparing cervicitis against HSIL, expression of B7-H6 in these infiltrating mononuclear cells was higher in the cervicitis group (*p* = 0.043). Results are shown in Figs. 5 and 6.

## Discussion

Immunohistochemical expression analyses of B7 family members have led to reports demonstrating the expression of B7-H6 at the cell surface and in the cytoplasm in several types of stressed or transformed cells. These reports include evidence of B7-H6 in the following pathological conditions: ovarian carcinoma, oral squamous carcinoma, non-Hodgkin lymphoma, astrocytoma, glioma, melanoma, atopic dermatitis, and liver tissue from patients with hepatitis B virus-related acute-on-chronic liver failure, among others. Interestingly, the severity of these diseases has been positively correlated with the presence of B7-H6; in some cases, a negative correlation between B7-H6 expression and overall survival has also been seen, as well as a positive correlation between disease progression and B7-H6 [9, 11, 15, 19–23]. In contrast, in some pathologies, such as in non-small cell lung cancer or gastric carcinoma, B7-H6 has shown limited value as a prognostic marker for the disease [24, 25].

Despite the fact that other B7 superfamily immune checkpoint molecules have already been explored in the microenvironment of cervical cancer, until now, there have been no reports examining B7-H6 expression in cervical intraepithelial lesions or cervical cancer [26–28]. The findings of our present study demonstrate for the first time that B7-H6 is not expressed in the abnormal epithelial cells of low-grade squamous intraepithelial lesions (LSIL); while, in contrast, when the stage of the lesion is more advanced, such as high-grade squamous intraepithelial lesion (HSIL) or both cancer types, squamous cervical carcinoma (SCC) or uterine cervical adenocarcinoma (UCAC), there is a positive correlation with the immunoreactive score (IRS) for immunohistochemical B7-H6 detection. These results match those observed in the aforementioned studies in other diseases, where the severity of the pathology was correlated with the expression of B7-H6.

When we analyzed the cancer specimens according to their histological classification, we interestingly found that there was an even higher B7-H6 IRS in the UCAC samples when compared with the SCC samples. Thus, taking into consideration the fact that keratinocyte surface expression of B7-H6 is reduced in SCC in comparison to UCAC, it is possible that this ligand could be being released from the cell surface by mechanisms unknown at this moment. Proteolytic cleavage, and soluble liberation of B7-H6 via exosomes, are mechanisms that have been reported in different conditions such as sepsis or during the development of normal human pregnancy [17, 22, 29]. Also, in some malignant tumors, soluble B7-H6 has been proposed as an immune escape mechanism leading to dysfunction of NK cells via NKp30 receptor down-modulation [10, 11, 22, 30].

Under inflammatory conditions, it has been proposed that both forms (membrane and soluble) of B7-H6 could be regulated by different mechanisms including TRIF or Myd88-dependent TLR signaling pathways [9, 17]. Therefore, considering that cervical microbiota changes through progressive intraepithelial lesions advancing toward cancer, which is characterized by the presence of gram-negative bacteria, including *Fusobacterium spp* [31], we cannot discard the possibility that microbial diversity associated with either squamous cervical carcinoma or cervical adenocarcinoma might be able to activate different inflammatory pathways influencing consequently the production of soluble or membrane forms of B7-H6. However, at present no reports comparing microbiota in SCC and UCAC have been performed.

While it is well known that SCC and UCAC present different molecular profiles [32], as well as distinctive pathogenesis and clinical behavior, the current treatment regimens are still relatively the same for both histologically different cervical cancers; these conventionally involve surgery, radiotherapy, and chemotherapy. In line with the above, there is significant evidence that UCAC have a greater propensity to metastasis and have worse prognosis, than SCC [6, 33]. Thus, it is feasible to speculate that different expression patterns of B7-H6 might be responsible, in part, for some of these differences. Indeed, ongoing experiments of our group, for example, show that B7-H6 expression is higher in HeLa cells (an uterine cervical adenocarcinoma cell line), than in SiHa (a squamous cervical carcinoma cell line). Differences in B7-H6 expression, then, could be important for the design of unique patient-specific treatment protocols.

Aside from its role in delivering a stimulatory signal to NK cells, B7-H6 could also be acting as a signaling protein (for the tumor) per se. Indeed, it has been reported that B7-H6 has predicted intracellular signaling motifs, such as ITIM, SH2, and SH3 domains [34]. More recently, other studies have reported that the knockdown of B7-H6 leads to the inhibition of cell proliferation, migration and promotes apoptosis in triple negative breast cancer cells, non-Hodgkin lymphoma and glioma cells, suggesting important biological roles executed by this ligand [9, 15, 16, 35]. Therefore, it will be interesting to examine if the expression of B7-H6, particularly in UCAC, confers additional benefits/signals to the tumor cells that might influence biological processes, such as proliferation and migration. If so, this might explain in part why cervical adenocarcinomas have a worse prognosis than squamous cell carcinomas.

Regarding the expression of B7-H6 in cells other than the abnormal/transformed epithelial cells, our current findings are consistent with earlier findings of B7-H6 expression in immune cells, such as monocytes and neutrophils, under inflammatory conditions [17, 20]. Our results corroborate that an inflammatory environment, such as found in cervicitis or during the progression to cervical cancer [36, 37], can be associated with the expression of B7-H6 in immune cells. In our work, B7-H6 was found in cells infiltrating the stroma; other groups have reported similarly that B7-H6 is over-expressed in myeloid-derived cells in the dermis of atopic dermatitis lesions, as well as in inflammatory cells in the tissue stroma of oral squamous carcinoma [20, 23].

Surprisingly, B7-H6 was found in both the cytoplasm and membrane of plasma cells; moreover, the B7-H6 IRS in plasma cells was higher in concert with the advance of disease stage (significantly positively correlated). It is interesting to note that a previous study, in a transgenic mouse model, reported that B7h (another member of the growing B7 family, ICOSL) on the plasma cell surface drives an increase in the number of plasma cells secreting antigen-specific, high affinity, class-switched antibodies, as well as a corresponding increased in serum concentrations of antigen-specific antibodies [38]. Thus, we cannot discard the possibility that B7-H6 might promote similar roles in plasma cells in inflammatory conditions of the cervix; however, to elucidate the exact function of B7-H6 expressed by plasma cells infiltrating the stroma, future experiments will be required in order to address this speculation.

One major limitation of this study was the absence of well-defined control tissues. Clearly, due to ethical guidelines, biopsies of the healthy cervix could not be obtained. For this reason, and in order to have the closest to what we considered a good and representative control, this study utilized biopsies of the cervix removed due to different causes, such as leiomyoma or prolapsed bladder surgery. All of these “control” samples presented cervicitis, and in 30% of these biopsies we observed delimited B7-H6 staining in the parabasal layer. Previous studies have reported B7-H6 expression in the basal epidermis of normal tissue; this is in contrast to atopic dermatitis lesions, where B7-H6 positivity was found in suprabasal layers [23]. However, we do not know yet whether this parabasal staining in our “control” tissues is analogous to the pattern that is expected to find in the normal cervical epithelia.

In summary, our results report for the first time the positive correlation between B7-H6 expression and stage during the natural history of cervical cancer. Certainly, these results need to be accompanied by further functional experiments, in order to elucidate the precise biological role of this molecule during the progression of cervical cancer. With this in mind, the differential expression pattern of B7-H6 between SCC and UCAC might prove to be a useful immunomarker that might be considered as a candidate for the design of treatment approaches accordingly to the histological type of this cancer, which still remains as the third most deadly cancer among Mexican women.

## Conclusions

In conclusion, our work highlights that B7-H6 staining (scored as IRS) in abnormal/transformed epithelial cells is positively correlated with the progression of cervical cancer. Apart from epithelial cells, B7-H6 was also found in infiltrating mononuclear cells; specifically, B7-H6 was consistently expressed by plasma cells infiltrated in all groups. Finally the highest immunoreactive score for B7-H6 were observed in tumor cells of uterine cervical adenocarcinoma, suggesting B7-H6 as a possible immunotherapeutic target that could be address in the treatment for this type of cervical cancer.

## Methods

### Specimens

A total of 62 formalin-fixed, paraffinized cervical biopsies obtained from the Hospital General de Occidente (Secretaría de Salud Jalisco) were analyzed. The following biopsies were collected: ten from women with cervicitis (biopsied for causes other than lesions or cervical cancer), fourteen from patients with low-grade squamous intraepithelial lesion (LSIL), fourteen from patients with high-grade squamous intraepithelial lesion (HSIL), fourteen from patients with squamous cervical carcinoma (SCC) and ten from patients with uterine cervical adenocarcinoma (UCAC). The use of these biopsies was accompanied by letters of informed consent and institutional approval (detailed below). Mean ages and standard deviation of the different groups were as follows: cervicitis (49 ± 8); LSIL (32 ± 7); HSIL (35 ± 8); SCC (45 ± 7); UCAC (46 ± 12). Cervical tissues were independently evaluated by two different pathologists. After the first evaluation, tissues were further evaluated by a senior pathologist to confirm the first diagnosis; slides that did not match with the previous diagnosis were discarded.

### Immunohistochemistry for B7-H6

Using the paraffin-embedded cervical biopsies, 2 μm thick sections were cut. Sections were de-waxed with xylene and rehydrated with ethanol and finally water. Antigen retrieval was performed using citrate buffer pH 6, 95 °C for 25 min. Hydrogen peroxide (3–4%) was applied to block endogenous peroxidase and then rinsed with TBS; after washing, sections were incubated with primary rabbit anti-human B7-H6 polyclonal antibody (Abcam, Cambridge, MA, catalog number 121794), diluted 1:250 in antibody diluent from Leica for 35 min. Sections were rinsed with TBS and then incubated with post-primary antibody solution for 10 min, then again rinsed and horseradish peroxidase-labeled IgG anti-rabbit was applied for 15 min. Then, a solution containing 66 mM 3,3′-diaminobenzidine tetrahydrochloride hydrate and other stabilizer solutions containing (≤0.1%) hydrogen peroxide were applied for 3 min; subsequently, sections were rinsed with water and stained with hematoxylin < 0.1% 4 min. All steps were performed using the BOND-MAX Automated IHC/ISH Stainer (Leica Biosystems).

### Evaluation of immunohistochemical staining

Monitoring of the immunohistochemical staining was analyzed and photographed using a Zeiss Axio Imager 2 Research Microscope.

Staining for B7-H6 was reported in accordance with the immunoreactive score (IRS) system [39], which ranged from 0 (100% negative tumor cells) to 12 (100% strong staining tumor cells), as shown in Table 1.

**Table 1:** Immunoreactive score (IRS) system. IRS = (A x B)

A (% positive cells)	B (intensity)
**0 = no positive cells**	0 = no color reaction
**1 < 10% of positive cells**	1 = mild reaction
**2 = 10–50% of positive cells**	2 = moderate reaction
**3 = 51–80% of positive cells**	3 = intense reaction
**4 > 80% of positive cells**	

### Statistical analyses

Statistical analyses were performed using IBM SPSS Statistics 23 software. First, the Shapiro-Wilks test was performed to identify the distribution of the population. Due to the non-parametric distribution, the Kruskal-Wallis and U-Mann Whitney tests were applied. The Spearman correlation test was used to identify correlations among groups. Results were considered statistically significant when *p* ≤ 0.05.

## Data Availability

The datasets used and/or analyzed during the current study are available from the corresponding author on reasonable request.

## Abbreviations

HPV: Human papillomavirus
HSIL: High-grade squamous intraepithelial lesion
IRS: Immunoreactive score
LSIL: Low-grade squamous intraepithelial lesion
SCC: Squamous cervical carcinoma
UCAC: Uterine cervical adenocarcinoma

## References

[1] Bray F, Ferlay J, Soerjomataram I, Siegel RL, Torre LA, Jemal A (2018). Global cancer statistics 2018: GLOBOCAN estimates of incidence and mortality worldwide for 36 cancers in 185 countries. CA Cancer J Clin.

[2] Song D, Li H, Li H, Dai J (2015). Effect of human papillomavirus infection on the immune system and its role in the course of cervical cancer. Oncol Lett.

[3] Winer RL, Hughes JP, Feng Q, Xi LF, Cherne S, O'Reilly S, Kiviat NB, Koutsky LA (2011). Early natural history of incident, type-specific human papillomavirus infections in newly sexually active young women. Cancer Epidemiol Biomarkers Prev.

[4] Kumar N (2016). Cervical cancer; a nightmare for womanhood: review o f recent advances. Women’s Health Gynaecol.

[5] Zur Hausen H (2002). Papillomaviruses and cancer: from basic studies to clinical application. Nat Rev Cancer.

[6] Gadducci A, Guerrieri ME, Cosio S (2019). Adenocarcinoma of the uterine cervix: pathologic features, treatment options, clinical outcome and prognostic variables. Crit Rev Oncol Hematol.

[7] Waldhauer I, Steinle A (2008). NK cells and cancer immunosurveillance. Oncogene.

[8] Garcia-Iglesias T, del Toro-Arreola A, Albarran-Somoza B, del Toro-Arreola S, Sanchez-Hernandez PE, Ramirez-Dueñas MG, Balderas-Peña LMA, Bravo-Cuellar A, Ortiz-Lazareno PC, Daneri-Navarro A (2009). Low NKp30, NKp46 and NKG2D expression and reduced cytotoxic activity on NK cells in cervical cancer and precursor lesions. BMC Cancer.

[9] Che F, Xie X, Wang L, Su Q, Jia F, Ye Y, Zang L, Wang J, Li H, Quan Y (2018). B7-H6 expression is induced by lipopolysaccharide and facilitates cancer invasion and metastasis in human gliomas. Int Immunopharmacol.

[10] Semeraro M, Rusakiewicz S, Minard-Colin V, Delahaye NF, Enot D, Vély F, Marabelle A, Papoular B, Piperoglou C, Ponzoni M (2015). Clinical impact of the NKp30/B7-H6 axis in high-risk neuroblastoma patients. Sci Transl Med.

[11] Pesce S, Tabellini G, Cantoni C, Patrizi O, Coltrini D, Rampinelli F, Matta J, Vivier E, Moretta A, Parolini S (2015). B7-H6-mediated downregulation of NKp30 in NK cells contributes to ovarian carcinoma immune escape. Oncoimmunology.

[12] Cao G, Wang J, Zheng X, Wei H, Tian Z, Sun R (2015). Tumor therapeutics work as stress inducers to enhance tumor sensitivity to natural killer (NK) cell cytolysis by up-regulating NKp30 ligand B7-H6. J Biol Chem.

[13] Zou Yong, Bao Junjie, Pan Xingfei, Lu Ying, Liao Sihong, Wang Xicheng, Wang Guoying, Lin Dongjun (2015). NKP30-B7-H6 Interaction Aggravates Hepatocyte Damage through Up-Regulation of Interleukin-32 Expression in Hepatitis B Virus-Related Acute-On-Chronic Liver Failure. PLOS ONE.

[14] Brandt CS, Baratin M, Yi EC, Kennedy J, Gao Z, Fox B, Haldeman B, Ostrander CD, Kaifu T, Chabannon C (2009). The B7 family member B7-H6 is a tumor cell ligand for the activating natural killer cell receptor NKp30 in humans. J Exp Med.

[15] Wu F, Wang J, Ke X (2016). Knockdown of B7-H6 inhibits tumor progression and enhances chemosensitivity in B-cell non-Hodgkin lymphoma. Int J Oncol.

[16] Zhang B, Sun J, Yao X, Li J, Tu Y, Yao F, Sun S (2018). Knockdown of B7H6 inhibits tumor progression in triple-negative breast cancer. Oncol Lett.

[17] Matta J, Baratin M, Chiche L, Forel J-M, Cognet C, Thomas G, Farnarier C, Piperoglou C, Papazian L, Chaussabel D (2013). Induction of B7-H6, a ligand for the natural killer cell–activating receptor NKp30, in inflammatory conditions. Blood.

[18] Semeraro M. Neuroblastoma and gastrointestinal stromal tumor as a target for natural killer lymphocytes : the role of ncr3/nkp30. In: tel.archivesouvertes. fr. Université Paris Sud-Paris XI.2014. https://tel.archives-ouvertes.fr/tel-01083693/document. Accesed 12 Mar 2019.

[19] Zhou Y, Xu Y, Chen L, Xu B, Wu C, Jiang J (2015). B7-H6 expression correlates with cancer progression and patient’s survival in human ovarian cancer. Int J Clin Exp Pathol.

[20] Wang J, Jin X, Liu J, Zhao K, Xu H, Wen J, Jiang L, Zeng X, Li J, Chen Q (2017). The prognostic value of B7-H6 protein expression in human oral squamous cell carcinoma. J Oral Pathol Med.

[21] Chretien A-S, Fauriat C, Orlanducci F, Rey J, Borg GB, Gautherot E, Granjeaud S, Demerle C, Hamel J-F, Cerwenka A (2017). NKp30 expression is a prognostic immune biomarker for stratification of patients with intermediate-risk acute myeloid leukemia. Oncotarget.

[22] Schlecker E, Fiegler N, Arnold A, Altevogt P, Rose-John S, Moldenhauer G, Sucker A, Paschen A, Von Strandmann EP, Textor S (2014). Metalloprotease-mediated tumor cell shedding of B7-H6, the ligand of the natural killer cell–activating receptor NKp30. Cancer Res.

[23] Salimi M, Xue L, Jolin H, Hardman C, Cousins DJ, McKenzie AN, Ogg GS (2016). Group 2 innate lymphoid cells express functional NKp30 receptor inducing type 2 cytokine production. J Immunol.

[24] Zhang X, Zhang G, Qin Y, Bai R, Huang J (2014). B7-H6 expression in non-small cell lung cancers. Int J Clin Exp Pathol.

[25] Chen X-J, Shen J, Zhang G-B, Chen W-C (2014). B7-H6 protein expression has no prognostic significance in human gastric carcinoma. Pathol Oncol Res.

[26] Han S, Wang Y, Shi X, Zong L, Liu L, Zhang J, Qian Q, Jin J, Ma Y, Cui B (2018). Negative roles of B7-H3 and B7-H4 in the microenvironment of cervical cancer. Exp Cell Res.

[27] Tao J, Dai J, Hou S (2017). Association between B7-H1 and cervical cancer: B7-H1 impairs the immune response in human cervical cancer cells. Exp Ther Med.

[28] Kosmaczewska A, Bocko D, Ciszak L, Wlodarska-Polinska I, Kornafel J, Szteblich A, Masternak A, Frydecka I (2012). Dysregulated expression of both the costimulatory CD28 and inhibitory CTLA-4 molecules in PB T cells of advanced cervical cancer patients suggests systemic immunosuppression related to disease progression. Pathol Oncol Res.

[29] Gutierrez-Franco J, Hernandez-Gutierrez R, Bueno-Topete MR, Haramati J, Navarro-Hernandez RE, Escarra-Senmarti M, Vega-Magaña N, del Toro-Arreola A, Pereira-Suarez AL, del Toro-Arreola S (2018). Characterization of B7H6, an endogenous ligand for the NK cell activating receptor NKp30, reveals the identity of two different soluble isoforms during normal human pregnancy. Immunobiology.

[30] Delahaye NF, Rusakiewicz S, Martins I, Ménard C, Roux S, Lyonnet L, Paul P, Sarabi M, Chaput N, Semeraro M (2011). Alternatively spliced NKp30 isoforms affect the prognosis of gastrointestinal stromal tumors. Nat Med.

[31] Audirac-Chalifour Astride, Torres-Poveda Kirvis, Bahena-Román Margarita, Téllez-Sosa Juan, Martínez-Barnetche Jesús, Cortina-Ceballos Bernardo, López-Estrada Guillermina, Delgado-Romero Karina, Burguete-García Ana I., Cantú David, García-Carrancá Alejandro, Madrid-Marina Vicente (2016). Cervical Microbiome and Cytokine Profile at Various Stages of Cervical Cancer: A Pilot Study. PLOS ONE.

[32] Wright AA, Howitt BE, Myers AP, Dahlberg SE, Palescandolo E, Van Hummelen P, MacConaill LE, Shoni M, Wagle N, Jones RT (2013). Oncogenic mutations in cervical cancer: genomic differences between adenocarcinomas and squamous cell carcinomas of the cervix. Cancer.

[33] Samuels S, Ferns D, Meijer D, Van Straalen J, Buist M, Zijlmans H, Kenter G, Jordanova E (2015). High levels of soluble MICA are significantly related to increased disease-free and disease-specific survival in patients with cervical adenocarcinoma. Tissue Antigens.

[34] Kaifu T, Escalière B, Gastinel LN, Vivier E, Baratin M (2011). B7-H6/NKp30 interaction: a mechanism of alerting NK cells against tumors. Cell Mol Life Sci.

[35] Jiang T, Wu W, Zhang H, Zhang X, Zhang D, Wang Q, Huang L, Wang Y, Hang C (2017). High expression of B7-H6 in human glioma tissues promotes tumor progression. Oncotarget.

[36] Zheng R-r, Huang M, Jin C, Wang H-c, Yu J-t, Zeng L-c, Zheng F-y, Lin F (2016). Cervical cancer systemic inflammation score: a novel predictor of prognosis. Oncotarget.

[37] Parida S, Mandal M (2014). Inflammation induced by human papillomavirus in cervical cancer and its implication in prevention. Eur J Cancer Prev.

[38] Larimore K, Liang L, Bakkour S, William CS (2012). B7h-expressing dendritic cells and plasma B cells mediate distinct outcomes of ICOS costimulation in T cell-dependent antibody responses. BMC Immunol.

[39] Remmele W, Stegner HE (1987). Recommendation for uniform definition of an immunoreactive score (IRS) for immunohistochemical estrogen receptor detection (ER-ICA) in breast cancer tissue. Pathologe.

